# Editorial: Emerging talents in cancer immunity and immunotherapy: 2022

**DOI:** 10.3389/fimmu.2023.1311519

**Published:** 2023-10-31

**Authors:** Markus Maeurer

**Affiliations:** ^1^ Immunotherapy/ImmunoSurgery, Champalimaud Foundation, Lisboa, Portugal; ^2^ I Medical Clinic Johannes Gutenberg University Mainz, Mainz, Germany

**Keywords:** T-cell, education, immunotherapy, tumor, immune-response

A Research Topic produced by young talents in immunotherapy sounded like a great opportunity to encourage young scientists and physician-scientists to submit their best work, an opportunity to gain international recognition, as well as recognition from their immediate peers. As I embarked upon writing this piece, I felt that I may have to defend the existence of the series. Why a dedicated Research Topic for young career scientists? If individuals produce excellent results with high quality data sets, would they not survive within the evolution of the standard selection process and publish good work anyway? Nevertheless, a particular space for young talents in immunotherapy was created. A ´talent´ was originally a unit of currency in the Roman and Greek empires. What is the currency that youth brings to the table, and why do we dedicate a particular Research Topic to young talents in the highly competitive and fast growing field of immunotherapy? If we turn around, do individuals ´with talent´ find science, particularly medicine and immunotherapy, sufficiently attractive to invest their talents? I would like to mention here a personal encounter that I will never forget. The CIMT organization (association for cancer immunotherapy, www.cimt.eu) organized a phantastic summer school 2022 (CIMT Summer School for early - career cancer immunologists). The purpose of this summer school was not only the training and exchange of science, but also contact and encounters between academic teachers and young scientists, including physician-scientists. Two events were remarkable. First, ´´ask the professor´´—a conversation that simply may not take place in the structured working relationship at the homebase. Questions concerning hierarchy and a still male-dominated medical structure came up, as well as the challenges of living in cities, where salaries for young career scientists or physicians are hardly sufficient to support a family. However, the second point was more alarming: ´Who would like to become a leader?´ As far as I remember, among 30 people or so, only 2 or 3 individuals raised their hands. Where is the hunger to be a leader in this field? Is it so unattractive to become a leader in immunotherapy? The participants were smart, diligent, competent, and knew their field very well. One of the most frequent answers as to why an individual would not aspire to a leading role was the instability and rather unpredictable nature of career pathways, as well as the ever-present pressure to find resources for a research group and the responsibility to pay coworkers’ salaries. Funding is unpredictive in certain research areas. Most participants were not prepared to go through the situation of letting go coworkers due to insufficient funding. Most of the summer school participants experience this directly or indirectly via their peers. Hsiao and coworkers (1) describe, in painstaking detail in their visionary piece, the ´success and the next generation of physician scientists´ based on the description of Milewicz, comparing the physician-scientist career path to an assembly line, or rather, a ´leaky pipeline´ (2). The journey tends to start with a high-powered MD-PhD program, with some losses during that journey. Some individuals ultimately flow along the ´pipeline´ into the interim safe haven of an R01 grant if they live in the US—or to something equivalent if they live across the pond. What is the career path of young talents, particularly physician-scientists, in immunotherapy? Only 12% of individuals in training end up with a position that is split between 20% clinical work and 80% research (3).

In terms of funding for research and clinical training, would it be helpful to leave the ´standard pipeline´ of the standard career path? Hsiao suggested a ´tree model´ with a strong trunk in excellent research (and clinical activity for physician-scientists), with ramifications for different pathways in industry and private and public institutions, including the building of new clinical structures, the creation of intellectual property, and the creation of novel diagnostic or therapeutic platforms, which may also include start-up companies set up by individuals with excellent ideas, zest, and resilience, to put products on the market (1). International competitions in public and private institutions employ science competitions, e.g., the ´Feed the Next Billion´ (XPRIZE) or the Earthshot Prize (4). This narrow window of opportunity is an indicator of the strong, unmet need for solutions in innovation and the presence of young talents in the market. Such singular events may certainly change the professional life of a small number of individuals; however, they are not the norm. Different kinds of ´talents´ may include the flexibility of individuals and the ability and willingness to switch career paths during the active working life; other distinct ´talents´ may in part lie in swift adaptability and the ability to incorporate new knowledge in the career portfolio. Resilience and adaptation appear to be some of the ingredients needed to make a career in the fast growing and diverse areas of immunotherapy. For instance, the advent of gene-edited immunotherapeutic products will certainly shape a distinct field of immunotherapy. More than 200 gene therapeutic products are currently in phase 2 or 3 clinical trials; while this is happening for rare genetic disease (5), genetically engineered immune cells are mainstream drugs for patients with certain forms of hematopoietic cancers, and the treatment of patients with solid cancer still awaits the silver lining of success. The clinical use of gene-edited immune cells for the treatment of patients with solid cancer is still in its infancy and is currently being explored (6). Immunotherapy is practised within multi-disciplinary teams and requires cross-fertilizing knowledge and preclinical and clinical teamwork. Talents need an ecosystem—this may be a local ecosystem in public or private non-profit organizations, in academia, or in commercial entities. If these ecosystems are not locally available, networks among different institutions may substitute and carry tangible value with regard to training and combining pre-clinical and clinical knowledge, which is in part also reflected by the success of the immunotherapeutic networks SITC (www.SITC.org) and CIMT (www.CIMT.eu). Both of these networks started with humble beginnings and developed into international, strong networks with attractive training programs for young career scientists and physician-scientists. Immunotherapy is practised *within* programs but *from people*. Moreover, the field of immunotherapy certainly requires dedicated, passionate individuals who are willing to invest their talents into this fascinating, fast-growing field after a rigorous and strong training program in science and clinical immunology. Perhaps this Research Topic could signal the unmet need to recruit dedicated and educated individuals from different areas of immunotherapy—it certainly shows that talented individuals are interested in joining the exciting field of immunotherapy; private, public, and commercial entities may consider creating a win-win situation for the patient(s), the young talent, and the host institution.

## Author contributions

MM: Writing – original draft.

## References

[1] Hsiao CJ Fresquez AM Christophers B. Success and the next generation of physician-scientists. J Clin Transl Sci (2020) 4:477–9. doi: 10.1017/cts.2020.491 PMC8057406 33948222

[2] Milewicz DM Lorenz RG Dermody TS Brass LF National Association of MD-PhD Programs Executive Committee. Rescuing the physician-scientist workforce: the time for action is now. J Clin Invest (2015) 125(10):3742–7. doi: 10.1172/JCI84170 PMC4607120 26426074

[3] Ahn J CD W Man L-X Greeley SAW Shea JA. Educating future leaders of medical research: analysis of student opinions and goals from the MD–PhD SAGE (Students’ Attitudes, Goals, and Education) survey. Acad Med (2007) 82(7):633–45. doi: 10.1097/ACM.0b013e318065b907 17595558

[4] Tay A. (Interviewer) Science competitions can help to catapult your science into the real world. Nature (2022) 603:957–9. doi: 10.1038/d41586-022-00581-x 35228736

[5] Editorial. Gene therapies should be for all. Nat Med (2021) 27:1311. doi: 10.1038/s41591-021-01481-9 34385703

[6] Qi Cai Q Warren S Pietrobon V Maeurer M Qi LS Lu TK. Building smart CAR T cell therapies: The path to overcome current challenges. Cancer Cell (2023) 41(10): 1689–95. doi: 10.1016/j.ccell.2023.08.011 37714150

